# “Struggling for independence”: The meaning of being an oldest old man in a rural area. Interpretation of oldest old men's narrations

**DOI:** 10.3402/qhw.v9.23088

**Published:** 2014-02-13

**Authors:** Tove Mentsen Ness, Ove Hellzen, Ingela Enmarker

**Affiliations:** 1 Department of Nursing, Mid-Sweden University, Östersund, Sweden; 2 Department of Health Sciences, Nord-Trøndelag University College, Namsos, Norway; 3 Centre of Care Research, Steinkjer, Mid-Norway

**Keywords:** Oldest old, phenomenological hermeneutics, rural

## Abstract

The amount of older people receiving home nursing care is increasing; in rural areas, they are at additional risk because of the distance between people and health care facilities. No specific studies have been found about oldest old men living alone and receiving home nursing care and the meaning of living alone in one's own home. The aim of this study was therefore to illuminate the meaning of being an oldest old man living alone in a rural area and receiving home nursing care. A sample of 12 oldest old men living in rural areas in the middle of Norway was chosen for this study. Narrative interviews were conducted, and data were analyzed using the phenomenological hermeneutical method. After a naïve reading and a structural analysis of the text, we identified three themes: *feelings of insufficiency in everyday life, finding hope in life*, and *feeling reconciliation with life*. The comprehensive understanding suggested that being an oldest old man living alone in a rural area means a struggle between a dependent existence and a desire to be independent. Living in the tension between independence and dependency is a complex emotional situation where one is trying to accept the consequences of life and loss—reconciling the wish to live with the fact that life will come to an end.

Aging is a multidimensional concept, reflecting a wide range of biological, psychological and social changes in life (Johnson, Bengtson, Coleman, & Kirkwood, 2005). Older people are at higher risk of not only increasing health problems but also of social isolation through losses of spouse and close family members and friends. Social isolation refers to objective physical separation from other people, such as living alone or residing in a rural geographic area.

In recent decades, there has been a significant increase in the number of older people living alone in their own homes (Chandler, Williams, Maconachie, Collett, & Dodgeon, 2004). For example, in Norway, six out of ten persons over 80 years of age live alone (Andreassen, 2011). It may be argued that the idea of “home” needs to be clarified. In the Western world, “home” is built around some basic principles such as “home” being a social place including recognition and respect for traditional family hierarchies as well as presenting a sense of social and community security. Home is a place that represents social and physical security. The home environment may provide sick people with greater control over their lives and more comfort than, for example, medical institutions (Di Mola, 1997).

Remaining in their own homes is important to older people (Hammer, 1999). In Western countries, the number of older people receiving home nursing care is increasing, and the number of older people living at institutions is decreasing (Tarricone & Tsours, 2008). Older people living alone are also more likely to receive help from home nursing care, friends, and family (Otnes, 2011). In rural areas, they are at additional risk because of the distance between people and health care facilities, which can result in geographic isolation. According to Hinck (2004), valuable services to help older people at home include housekeeping and home maintenance, transportation, personal care, and management of medical conditions. To enhance the implementation of these services for older persons, a better understanding of what is important to them is needed. This study offers a narrative voice of rural community-living persons aged over 80 as a source for further learning about how they view and manage day-to-day living. No specific studies have been found about oldest old men living alone and receiving home nursing care and the meaning of living alone in one's own home. Therefore, the aim of this study was to illuminate the meaning of being an oldest old man living alone in a rural area and receiving home nursing care.

## Method

A qualitative approach was chosen for this study because when studying peoples’ experiences and seeking to understand their lives and worlds, it seems important to talk to them with the purpose of trying to understand the world from their point of view (Patton, 2002). This method applies an inductive style, focusing on individual meaning and the importance of rendering the complexity of a situation (Creswell, 2009). Therefore, the data interpreted in this study represent personal stories about personal experiences, which should be seen as unique and never generalized.

### Participants

A sample of 12 oldest old men, between 82 and 94 years of age, living in rural areas in the middle of Norway was chosen for this study. The informants lived in rural areas in eight different municipalities. Occupational background varied from farmers and fishermen to having an office job. Closeness to relatives varied, in distance and in degree of contact. All of the men lived alone and received home nursing care. Some had visits several times a day, others once a week. Most informants were widowers, but three informants had wives at a nursing home. To gain access to the informants, the first author contacted the health care leader in each municipality, who communicated the information to the home nursing care office. The home nursing care leader contacted possible informants and informed them about the study and arranged contact with those who were interested in participating. After that, the first author arranged the meeting, but the informant decided the time and place. Participants were informed about the purpose of the study verbally and in writing. This was done by the home nursing care contact and by the first author.

### Interviews

The first author conducted narrative interviews (Mishler, 1986) during the summer and autumn of 2012. Older men were asked the following questions regarding specific themes:

- “Please, can you tell me about your experience of receiving home nursing care?”
- “Please, can you tell me about how you experience being an old man today?”
- “Please, can you tell me about how you experience living alone?”

A story gives rich and important information about the person who narrates it. According to Sandelowski (1994), we are the stories we tell. To illuminate the meaning of being an oldest old man, narrative interviews could contribute to gathering more detail about this research phenomenon. During the interviews, clarifying questions were asked only when the interviewer did not understand, when she wanted the informants to develop their stories, and when deeper reflection was needed. The interviews were carried out in the informants’ own homes and lasted 98 to 150 minutes. The interviews were recorded and transcribed verbatim by the first author.

### Data analysis

Ricoeur (1976) describes interpretation of a text as the hinge between language and lived experience, with an ongoing dialectic movement between the parts and the whole of the text—between explanation and understanding. “To understand a text is to follow its movement from sense to reference: from what it says (its meaning), to what it talks about (its reference)” (Ricoeur, 1976, pp. 71–88). Interpretation includes three phases: naïve understanding, structural analysis, and comprehensive understanding (interpreted whole) (Lindseth & Norberg, 2004; Ricoeur, 1976). In the first phase, the transcribed interview texts were read separately several times in an open-minded manner to grasp their meaning as a whole. This reading gave a first, naïve understanding of each interview text—a naïve understanding of the meaning of the lived experience of being an oldest old, living alone in a rural area and receiving home nursing care. The interpretation continued with a structural analysis to explain the text, validate the naïve reading, and to identify and formulate subthemes. This thematic analysis began with the whole text from each interview set and worked through the text sentence by sentence, dividing the material into meaning units in accordance with the aim of the study and the naïve understanding. A meaning unit could be a part of the text of any length relating to the same meaning and depending on the shift in content. Each meaning unit was carefully condensed into everyday language and then abstracted. It was further reflected upon and compared, making it possible for structures to emerge and to group the abstracted meaning units into subthemes and themes. An example is given in Table I. In the third phase, the naïve understandings and the structural analysis of being an oldest old living alone in a rural area and receiving home nursing care were woven together to reach a comprehensive understanding of the whole and were reflected upon in relation to the authors’ pre-understanding, the research question, the context of the study, and relevant literature.

**Table I:** Example of the data analysis process.

Meaning unit	Condensed meaning unit	Sub theme	Theme
But I do think about it … maybe it has been for the best if I have died. They can do much more if they want … if they haven't had me to worry about, me who sit here alone …	Best if I have died. Not had someone to worry about, when alone.	Fearing becoming a burden	Feeling insufficiency in everyday life
I can die any minute now. The home nursing care can find me any day.	Can die any minute now.	Feeling insecure	Feeling insufficiency in everyday life
Of course it gets a little lonely, but then I take some trips to the nursing home … even if it's nothing special. Then I can stay there and drink coffee with them. That is so nice.	It gets lonely, but can get out and meet others. That's nice.	Experience new possibilities	Finding hope in life

### Ethical considerations

The informants were thoroughly briefed beforehand and guaranteed confidentiality, and no individual characteristics were to be disclosed in reporting the results. The informants’ names were excluded from transcriptions, which were kept in a locked facility to which only the first author had access. Each informant gave his informed consent before he participated in the study. Permission for research was granted by the Norwegian Social Science Data Services (No. 28728) and carried out in accordance with the Declaration of Helsinki (WMA, 2008).

## Findings

### Naïve reading

The first phase of interpretation included several naïve readings of the interview text, which resulted in a naïve first understanding of the text as a whole. The oldest old men experienced loneliness and insecurity in their everyday life. During the times when they were receiving help from home nursing care or family, they felt safe. On the one hand, they felt gratitude toward family and the home nursing care personnel for their practical and psychological support, but on the other hand, their impairments made them feel afraid of being seen as burdensome by family or home nurses. Receiving help relieved the men from loneliness and provided a sense of togetherness. Despite their losses, these men felt that they had the ability to handle the everyday activities connected to life at home. Contact with others made them feel less dependent and gave meaning to their lives as old men living alone. This naïve understanding guided the next phase of interpretation, thematic structural analysis, which, in turn, aimed to validate the naïve understanding. All the text containing information on support was divided into meaningful units (that is, units of text that contain the same meaning). Units of meaning were then condensed and abstracted into subthemes and themes.

### Structural analysis

The results of this study are shown in three themes and seven subthemes. The themes summarize oldest olds’ views of living alone in their own home. The first theme, “feelings of insufficiency in everyday life,” has three subthemes (“feeling insecure,” “being grateful,” and “fear of becoming a burden”) that point to the importance of oldest old men receiving support from formal and informal care during their time at home. The second theme, “finding hope in life,” has one subtheme (“experience new possibilities”) that represents oldest olds’ ability to handle their situations in creative ways. “Feeling reconciliation with life,” the third theme, has three subthemes (“accepting losses in life,” “handling a new life situation,” and “experience meaning”) that show the old men's feelings and their will to live (that is, to have courage to live).

#### Theme 1: Feelings of insufficiency in everyday life

This theme highlights the insecurity that oldest old persons feel when living at home in their own homes. A sense of insecurity is reflected in the unease that oldest old persons feel at home; this worry declines when care providers or family visit them—visits for which they were grateful.

*Feeling insecure* means that the old men feel like they had been left alone with declining social contacts. After their wives’ deaths, visits from the home nursing care providers and their family were their main human contact. The oldest old men feel insecurity because of decreasing health, increasing age, and because of the fact that they are alone. They are all concerned that something may happen to them. “I can die any minute now. The home nursing care can find me any day.”

After falling, they have had several experiences with long waits before receiving home nursing care assistance. It is important to oldest old men to make adjustment in their everyday life, one example is not going very far from their house in case their safety alarm would not work.

*Being grateful* means thankfulness toward their caregivers (family and home nursing care) because of the practical and psychological support given them. Even so, they try not to ask for help too often even if help is needed because they do not want to strain their relationship with their family and with home nursing care. “I think they all are good people, even though they are different. I wonder if they have picked the best people to become nurses.”

Oldest old men let the other partner in the relationship decide what to talk about. Through talking about what the other person is engaged in or by showing interest in the others’ topics of conversation, the oldest old men feel that their indebtedness decreases. Gratefulness makes old men ignore other peoples’ faults even if their values are different.

*Fearing becoming a burden* means that oldest old men fear becoming burdensome. They view caregivers’ jobs as demanding—perhaps because they had seen their wife or close relatives or friends being cared for while demented or physical impaired. Old men's fears of being dependent on care and of feeling loneliness are strong. They realize that their life situation and age may lead to a growing need for care and support in the future, which frightens them.

But I do think about it … maybe it would have been best if I had died. They could do much more if they wanted … if they didn't have me to worry about, me who sits here alone …

Oldest old men sometimes emphasize old peoples’ low value when needing help, and they do not want to become a burden to their caregivers as they have seen other old people become.

#### Theme 2: Finding hope in life

This theme refers to oldest old men's natural desire to function as independent persons; as old men, they have to redefine their lives and find new meaning in life. Searching for creative solutions decreases feelings of loneliness.

*Experience new possibilities* means that oldest old men saw new meaning in life and in influences on their lives even as their health was deteriorating. Some men still drive their cars and use them to dispel their loneliness and to seek out new social arenas.

If I don't want to sit alone, I can just take a little trip with my car … //… It means everything to get out, because it's there you experience everything. It makes me lively.

With decreasing health, being alone, and losing their driving license, several old men considered moving closer to the community's center. In that way, they could meet other people at the local café or in their new neighborhood and interact with others without using their car (for example, they could use an electric wheelchair). By considering such a move, the oldest old men were seeing new possibilities in their life as oldest old men living alone.

I think it's better to sit there with others, instead of sitting alone in our houses …//… You must have somebody to talk with, if not life gets a little difficult I think …//… You must get out, because you are dependent on being with others.

Seeing possibilities in life makes the old men feel less dependent and isolated. Facilitating for other people is important and cultivates feelings of community. For example, the nursing home represented a new possibility because it is a place they already have or have had a connection to through relatives, friends, or a deceased wife.

Of course it gets a little lonely, but then I take some trips to the nursing home … even if it's nothing special. Then I can stay there and drink coffee with them. That is so nice.

#### Theme 3: Feeling reconciliation with life

This theme reflects the oldest old men's will to live even as they become more and more fragile, impaired, and lonely.

*Accepting losses in life* means being reconciled with losses in life, especially the loss of a spouse, but also acceptance of a declining circle of acquaintances and of fragile health and body. The oldest old men accept loss as a natural consequence of getting old; therefore, they had to accept it. Positive thoughts and attitudes help these men to manage everyday life and not to dwell on their loneliness.

You can't focus on what you can't do; if you are thinking about that, you destroy your life …//… Even if you are alone, it's good to think about that you always can do what you want to do, and you don't have to consider anybody else. I couldn't do that before.

Looking back on life, the oldest old men feel content about how they lived theirs. Men highlighted that they now are debtless because they had contributed their share to their communities and their families.

*Handling a new life situation* means a struggle between having a positive outlook on life and having negative thoughts connected with isolation and loneliness. The old men state that negative thoughts always are lurking around the corner; these thoughts are mostly connected to their wives and the fact that they miss that companionship. “You have to stop thinking about that; if not, you get a little bit crazy.”

The oldest old men's declining health means that they cannot do all the things they want such as hunting, cutting wood, and hiking. By making adjustments and practicing appreciation, they see the openings in life and are able to handle new life situations. Going to bed earlier is a common way to shorten the day and feel less alone. Independence grows from a sense of not imposing on anyone and not demanding anything from family and friends, even though the old men appreciate the visits they receive.

*Experience meaning* relates to their relationship with nature, even if declining health means that the oldest old men cannot take the same trips as when they were young. Even if the oldest old men's inner wish is to walk in the company of man, for all men the reality is that they must find new meanings in life. Nature gives pleasure and meaning, and means nearness and a sense of wholeness.

So if I'm out in the garden I don't feel lonely and even if I'm only seeing the birds, it's enough company for me.

Filling their time with daily activities helps the men to experience meaning, and the fact that daily activities take more time than they did previously pleases them because this helps them to fill their time. Hobbies and books give meaning in life. Another important way to find meaning is to reflect on memories of earlier times. One example of this might be a sense of spiritual connection with a deceased wife.

### Comprehensive understanding

Being an oldest old man living alone in a rural area is a struggle between a dependent existence and a desire to be independent. The desire for independence forces these men to aspire to not burdening their relatives or formal care providers and, as much as possible, to handle everyday life on their own. However, declining health and increasing age mean dependency, making these men more dependent on relatives and/or home health care providers. This tension between independence and dependency encompasses a variety of emotions and experiences that are shaped by how men use inner resources to handle everyday life. Getting older means not having the same control over one's body and life choices; oldest old men want to have a sense of control over their lives by self-determination of what gives life meaning as an oldest old. The struggle between independence and dependency means living in a complex emotional situation and trying to accept the consequences of life and loss—reconciling the wish to live with the fact that life will soon come to an end.

## Discussion

The aim of this study was to illuminate the meaning of being an oldest old man living alone in a rural area and receiving home nursing care. From this analysis, the following three themes were revealed: *feelings of insufficiency in everyday life*, *finding hope in life* and *feeling reconciliation with life*. The main findings were related to the struggle between a dependent existence and a desire to be independent. This complex emotional situation means trying to accept the consequences of life with different losses and the wish to live, while concurrently reflecting on and being reconciled with the fact that life will soon come to an end.

Even if the oldest old men living at home appreciated, and for the most part were satisfied with, the practical and psychological support they were given by family or care providers, we found in the theme *feelings of insufficiency in everyday life* that they had a sense of dependency. This was reflected in their unease and their feelings of insecurity. This sense of dependency agrees with the findings of Burström, Brännström, Boman, and Strandberg (2011) and Forsman, Herberts, Nyqvist, Wahlbeck, and Shierenbeck (2013), who state that in later life, when health deteriorates, the possibilities for engaging in community life are limited and dependency on others increases. Research also suggests that living alone (Victor, Scambler, Bond, & Bowling, 2000), widowhood (Pinquart, 2003), functional dependence (Prieto-Flores, Forjaz, Fernandez-Mayoralas, Rojo-Perez, & Martinez-Martin, 2011), and lack of contact with friends and neighbors (Bondevik & Skogstad, 1998) are risk factors for loneliness. Loneliness appeared to be a factor for the oldest old men in our study. According to Ernst and Cacioppi (1999), loneliness, as a feeling of aloneness and being apart from others, is an unfavorable balance between actual and desired social contact. The oldest old men's wishes to be independent and not to burden their relatives or care providers led to loneliness in everyday life.

*Finding hope in life* helps the oldest old men to see and experience new possibilities in life and to cope with declining social relationships and loneliness. Still having opportunities to interact with others outside their homes helps them to cope with loneliness (*cf*. Pettigrew & Roberts, 2008). According to Aday, Kehoe, and Farney (2006), this may give the oldest old men a more positive strategy for strengthening positive life orientation. Even if they prefer being independent, men have a pragmatic view about receiving care (Roe, Whattman, Young, & Dimond, 2001). In our study, the oldest old men were searching for ways to interact with others outside the home (for example, visiting the nursing home). As a positive consequence, this creates a feeling of independence and fosters hope. It seems that as life's fragility emphasizes closeness to death but not defeat, an opening to life is also created (*cf*. Jaspers, 1970). Our findings are consistent with those of Hinck's (2004) study where oldest-old rural adults seemed to define their health by their ability to function rather than by recognition of their pain and other illness symptoms.

Even if getting old implies living with many losses (Fisher, Norberg, & Lundman, 2008; Graneheim & Lundman, 2009), the oldest old men in our study seemed to accept their losses and see new possibilities and meaning in their everyday lives. Using their memories helped them toward *feeling reconciliation with life*. Sometimes feelings of social isolation and loneliness may be dispelled by memories of the companionship of a deceased wife. Filling time with daily activities that take longer to complete than they did in younger days may also give meaning (*cf*. Birkeland & Natvig, 2009). According to Borglin, Jakobsson, Edberg, and Hallberg (2006), an important factor for judging quality of life in old age is the sense of meaning in existence, that is, the manner in which the individual's life is viewed, thoughts about death and dying, and the opportunity to tell one's story.

In Western culture, the final stage of life is filled with a sense of not really being important. Family members and professionals who assume older people cannot have a meaningful life because the “fruitful” and “productive” years are behind them sometimes reinforce this (Suri, 2010). However, one major source of meaning for older persons is the sense that others respect them. The search for meaning is an ongoing, lifelong process. It does not stop simply because an individual reaches the final stage of life. Human beings not only seek to understand their own existence, they seek meaning in their lives (Längle & Sykes, 2006).

According to Jaspers (1970), man's life is in continuous motion, which is split in antinomies. It is the antinomies that are the source of life's boundary situations (i.e., situations where one experiences one's own and existence's limits). Death is one of Jaspers’ (1970) defined boundary situations, by which he means humans’ enlightenment to finitude. Logically, the human condition means that we live and we die, and one's life is between those two poles. Life is constant movement, a trek that changes along the way. Therefore, humans must constantly reproduce values and transfigure life. Our interpretation points out that this is exactly what the oldest old men in our study do. They feel hope and reconcile with their life situation; they see new possibilities, such as creating new meanings in life that help them to manage life. Being an oldest old man inevitably means being near death.

Our findings indicate that the fear of becoming burdensome to others and of being dependent on others is strong. The oldest old men realize that their life situation and age may lead to a growing need for care and support. According to Strandberg, Norberg, and Jansson (2003), being dependent on others means considering one's dependency and the feeling of being burdensome. The wish to not be a burden and to maintain as much independence as possible is important for older persons’ dignity (Sundin, Bruce, Barremo, 2010). However, this feeling is not always associated with objective evidence of any impairment (Akazawa et al., 2010); they may feel burdensome even if they need little or no help from caregivers (Yamagishi et al., 2012).

Being dependent on care may cause guilt and shame, limited freedom, and feelings of being handicapped (Sundin, Bruce, Barremo, 2010). Other negative feelings are, according to Hellström and Sarvimäki (2007), feelings of resignation, of not being valued, and of having no influence. The fear of becoming burdensome because of growing dependency is a strong emotion. In rural areas, the oldest old people want to stay at home as long as possible, even if this means being mostly alone (Hinck, 2004). When suffering from long-term illness, those striving for independence place valuable meaning in everyday activities (Delmar et al., 2006). Our study indicates that the oldest old men tend to seek out others after the loss of their wives (*cf*. Van den Hoonaard, 2009). This tendency toward outgoing activities may be explained from a gender perspective, according to Russell (2007), who stresses that home has a different meaning to older men than to older women. Older men have worked outside the home, while women have spent much of their time in “the making of a home.” Therefore, they are more locality-bound to home than men. Our findings therefore suggest that oldest old men in rural areas want to stay at home as long as possible but only if they have significant others to which they can relate.

### Methodological considerations

It is possible to interpret text in different ways. Our findings constitute one of several possible interpretations (Ricoeur, 1976). The phenomenological perspective focuses on the person's lifeworld and lived experience, and thus it requires openness to the interviewee's experiences (Lindseth & Norberg, 2004). The gateway to this lifeworld runs through the narrative, with all its symbols and metaphors, and has to be interpreted by using explanation and understanding (Ricoeur, 1976). In the hermeneutic tradition, issues of trustworthiness are a matter of difficulty throughout the process, which aims to arrive at possible interpretations—not at finding the absolute truth. The first author carried out the initial analysis; however, all three authors have reflected on and have continuously and critically worked with the assessments until a consensus was reached. Our interpretation represents what we have found to be the most useful way of understanding this phenomenon. Therefore, this research offers one perspective, which may constitute a basis for further reflections about when the patient's expressions become the nurse's impression, that is, when the nurse is emotionally touched by the patient's expressions.

### Conclusion and implications for nursing practice

Based on the findings of this study, there is an extensive need to provide knowledge about the meaning of being an oldest old man, living alone in a rural area and receiving home nursing care. Such knowledge may bring to light health promotion strategies for how nursing care contributes to enhancing oldest old men's independence. The accelerating population in this age group cries out for more research aimed at better understanding the challenges associated with the final stage of life. Understanding the perspectives of this group of men may be a basis for developing future interventions to help them remain at home as long as possible (for example, by facilitating relations with significant others after the loss of a spouse).
